# Supplementary material for: Comparative Genomics Reveals Evidence of the Genome Reduction and Metabolic Potentials of Aliineobacillus hadale Isolated from Challenger Deep Sediment of the Mariana Trench
Source: Microorganisms. 2025 Jan 10;13(1):132. doi: 10.3390/microorganisms13010132 (PMC11767280; doi:10.3390/microorganisms13010132)

#Supplementary Figure S1: Neighbor-joining phylogenetic tree based on 16S rRNA genes. The numbers in parentheses are the NCBI accession numbers of the reference gene sequences. The scale unit length indicated a unit nucleotide position change rate of 0.1.

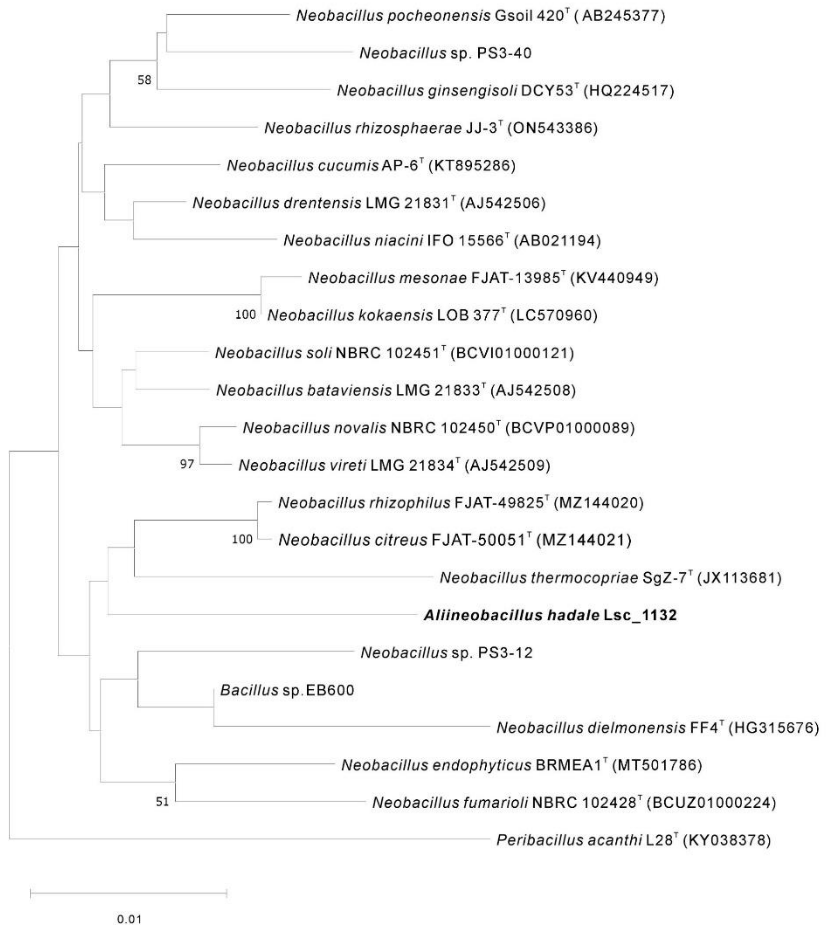

Supplement: Supplementary file 1 [file microorganisms-13-00132-s001.zip › Supplementary Figure S1.pdf]
