# Supplementary material for: Comparative Genomics Reveals Evidence of the Genome Reduction and Metabolic Potentials of Aliineobacillus hadale Isolated from Challenger Deep Sediment of the Mariana Trench
Source: Microorganisms. 2025 Jan 10;13(1):132. doi: 10.3390/microorganisms13010132 (PMC11767280; doi:10.3390/microorganisms13010132)

#Supplementary Figure S2: Venn diagram representation of shared and unique genes across the strains Lsc_1132^{T}, E600, PS3-12 and PS3-40.

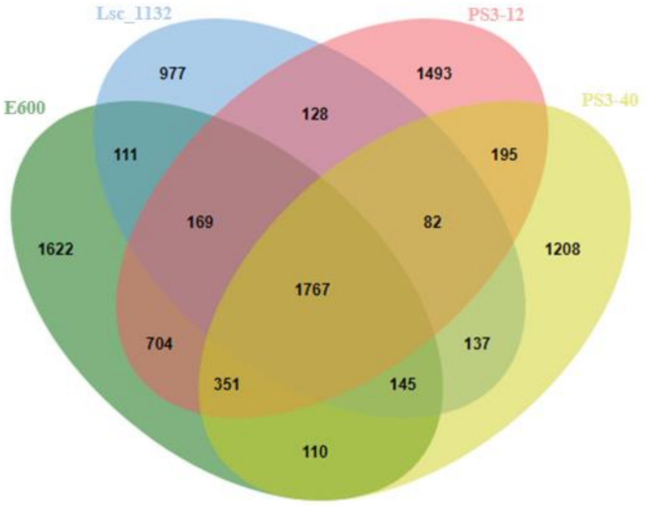

Supplement: Supplementary file 1 [file microorganisms-13-00132-s001.zip › Supplementary Figure S2.pdf]
