# Supplementary material for: Comparative Genomics Reveals Evidence of the Genome Reduction and Metabolic Potentials of Aliineobacillus hadale Isolated from Challenger Deep Sediment of the Mariana Trench
Source: Microorganisms. 2025 Jan 10;13(1):132. doi: 10.3390/microorganisms13010132 (PMC11767280; doi:10.3390/microorganisms13010132)

#Supplementary Figure S3: Graphical representation of the strain Lsc_1132[T] genomes. The outermost circle of the circus plot represents the genome size markers. The second and third circles denote different COG functional categories. The fourth circle indicates the locations of rRNA and tRNA genes. The fifth circle represents GC content, with outward red segments indicating regions where the GC content is above the genome-wide average, while inward blue segments indicate regions where the GC content is below the average. The innermost circle shows the GC skew values.

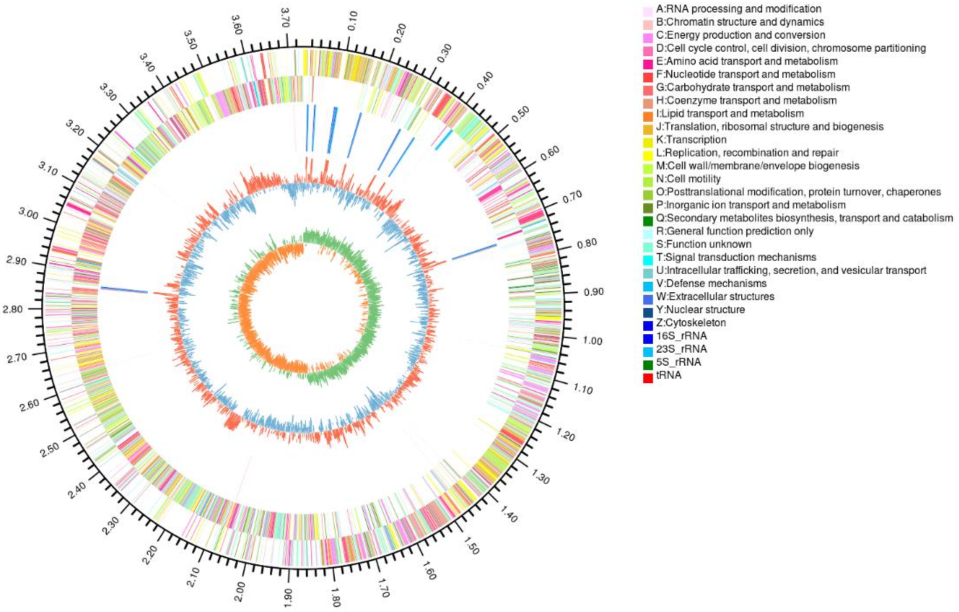

Supplement: Supplementary file 1 [file microorganisms-13-00132-s001.zip › Supplementary Figure S3.pdf]
