# Supplementary material for: Comparative Genomics Reveals Evidence of the Genome Reduction and Metabolic Potentials of Aliineobacillus hadale Isolated from Challenger Deep Sediment of the Mariana Trench
Source: Microorganisms. 2025 Jan 10;13(1):132. doi: 10.3390/microorganisms13010132 (PMC11767280; doi:10.3390/microorganisms13010132)
Supplement: Supplementary file 1 [file microorganisms-13-00132-s001.zip › Supplementary Tables and Figures.pdf]

Supplementary Tables:

Table S1: Characters of MicroPlate GEN III results of strain Lsc_1132^T.

Table S2: The fatty acid results of strain Lsc_1132^T.

Table S3: The sequence similarity of the 16S rRNA between strain Lsc_1132^T and its closely related strains.

Table S4: The values of ANIb and aligned percentage between strain Lsc_1132^T and closely related strains.

Table S5: The DDH values between strain Lsc_1132^T and closely related strains.

Table S6: The AAI values between strain Lsc_1132^T and closely related strains.

Table S7: The COG classification of strains Lsc_1132^T, *Bacillus* sp. EB600 and *Neobacillus* sp. PS3-12.

Table S8: Prediction of transporter proteins for strain Lsc_1132^T**.**

Table S9: The extracellular peptidases predicted in strain Lsc_1132^T.

Table S10: The strain-specific loss of KEGG number in strain Lsc_1132^T.

Supplementary Figures:

Figure S1: Neighbor-joining phylogenetic tree based on 16S rRNA genes.

Figure S2: Venn diagram representation of shared and unique genes across the strains Lsc_1132^T, E600, PS3-12 and PS3-40.

Figure S3: Graphical representation of the strain Lsc_1132^T genomes.
